# Serum Procalcitonin Level and SOFA Score at Discharge from the Intensive Care Unit Predict Post-Intensive Care Unit Mortality: A Prospective Study

**DOI:** 10.1371/journal.pone.0114007

**Published:** 2014-12-02

**Authors:** Yosuke Matsumura, Taka-aki Nakada, Ryuzo Abe, Taku Oshima, Shigeto Oda

**Affiliations:** Department of Emergency and Critical Care Medicine, Chiba University Graduate School of Medicine, Chiba city, Chiba, Japan; Bambino Gesù Children's Hospital, Italy

## Abstract

**Purpose:**

The final decision for discharge from the intensive care unit (ICU) is uncertain because it is made according to various patient parameters; however, it should be made on an objective evaluation. Previous reports have been inconsistent and unreliable in predicting post-ICU mortality. To identify predictive factors associated with post-ICU mortality, we analyzed physiological and laboratory data at ICU discharge.

**Methods:**

Patients admitted to our ICU between September 2012 and August 2013 and staying for critical care>2 days were included. Sequential Organ Failure Assessment (SOFA) score; systemic inflammatory response syndrome score; white blood cell count; and serum C reactive protein, procalcitonin (PCT), interleukin-6 (IL-6), lactate, albumin, and hemoglobin levels were recorded. The primary end point was 90-day mortality after ICU discharge. Two hundred eighteen patients were enrolled (195 survivors, 23 non-survivors).

**Results:**

Non-survivors presented a higher SOFA score and serum PCT, and IL-6 levels, as well as lower serum albumin and hemoglobin levels. Serum PCT, albumin, and SOFA score were associated with 90-day mortality in multiple logistic regression analysis. Hosmer-Lemeshow test showed chi-square value of 6.96, and P value of 0.54. The area under the curve (95% confidence interval) was 0.830 (0.771–0.890) for PCT, 0.688 (0.566–0.810) for albumin, 0.861 (0.796–0.927) for SOFA score, and increased to 0.913 (0.858–0.969) when these were combined. Serum PCT level at 0.57 ng/mL, serum albumin at 2.5 g/dL and SOFA score at 5.5 predict 90-day mortality, and high PCT, low albumin and high SOFA groups had significantly higher mortality. Serum PCT and SOFA score were significantly associated with survival days after ICU discharge in Cox regression analysis.

**Conclusions:**

Serum PCT level and SOFA score at ICU discharge predict post-ICU mortality and survival days after ICU discharge. The combination of these two and albumin level might enable accurate prediction.

## Introduction

Admission to the intensive care unit (ICU) is considered when patients need intensive treatment and monitoring that cannot be provided outside of the ICU [1]. Mortality rates of patients admitted to the ICU remain high [2], [3], and even if the patients survive during the ICU stay and are successfully discharged, these patients could have sequelae, including deteriorated cognitive or physical function (post-intensive care syndrome) [4] or poor outcomes after the ICU and hospital discharge [5]–[7]. Although institutional and regional characteristics might affect ICU discharge criteria, critically ill patients are generally considered suitable for discharge from the ICU when their physiological status has stabilized and the need for ICU monitoring and care is no longer necessary [1]. In a critical care setting, the basis of the final decision for discharge is uncertain, because it is made according to various physiological variables and laboratory data. However, the decision should be made on an objective evaluation. Persistent inflammation, immunosuppression, malnutrition, and catabolism are potentially associated with poor clinical outcome after discharge from the ICU [8].

Serum C reactive protein (CRP) levels at ICU discharge [9], [10], maximum CRP levels during the ICU stay [10], CRP/albumin ratio at ICU discharge [11], and heart rate at ICU discharge [12] have been reported to be predictive of post-ICU mortality. However, results have been inconsistent. Furthermore, these parameters could not predict post-ICU mortality accurately. To identify prognostic factors associated with 90-day mortality after ICU discharge, we prospectively analyzed physiological status and laboratory data collected at ICU discharge.

## Methods

This prospective, observational, follow-up study was performed in the ICU of a tertiary-care university hospital in Japan. The hospital has 835 beds, and the 22-bed medical-surgical ICU admits 1,600 to 1,800 critically ill patients per year. The ICU has 24/7 coverage by intensivists, but there is no “Step-down” unit. The ICU team comprises intensivists, physicians from various specialty departments, ICU nurses, an infection control team (comprising physicians, nurses, and clinical technicians), physical therapists (early mobilization, respiratory rehabilitation), clinical engineers (medical equipment management, operating blood purification), pharmacists (therapeutic drug monitoring and referring drug interactions), and clinical nutritionists (planning and evaluation for nutrition) and convenes a daily conference to decide on treatment plans.

Critically ill patients are treated so as to cure their acute organ failure during ICU stay. The ICU team decided to discharge from the ICU when the patient was stabilized through acute care and ICU-specific treatments were no longer needed for organ failure. Acute organ failure might shift to chronic failure; such as disturbance of consciousness or renal failure. ICU team usually decide to discharge the patient from the ICU if other organ functions are stable. ICU-specific treatment includes mechanical ventilation, intra-aortic balloon pumping, temporary pace-maker, extra-corporeal membranous oxygenation, ventricular assist device, renal replacement therapy, therapeutic hypothermia. Some artificial supports are performed after ICU discharge; ventilator in chronic lung disease or intermittent hemodialysis in chronic kidney disease. After considering ICU patient's status, emergency ICU admission of more severe patient, and availability of ICU and general ward, attending ICU physician eventually decide ICU discharge.

This study protocol was approved by The Institutional Review Board at Chiba University (ethic approval number, 1635), which waived written informed consent because of the observational nature of the study.

### Patients

Patients admitted to the medical-surgical ICU from September 2012 to August 2013 and stayed in critical care>2 days were selected. Patients aged <18 years were excluded. The total ICU admission during the study period was 1,723. Seventy-five patients (4.4%) died during the ICU stay and 1,648 (95.6%) survived to discharge. A total of 247 cases stayed in critical care>2 days. Of these, 23 patients were repeatedly admitted to the ICU, and we only included 224 patients' data from their initial ICU admission. Six patients were excluded for age. Thus, 218 patients were enrolled for analysis.

### Measurements

The following patient characteristics were recorded: age, sex, diagnosis on ICU admission, Acute Physiology and Chronic Health Evaluation (APACHE) II score [13], Sequential Organ Failure Assessment (SOFA) score [14], readmission cases, surgical cases, admission diagnosis, usage of mechanical ventilation, renal replacement therapy and vasopressor, antibiotic treatment during the ICU stay and at discharge, infection at death, nocturnal discharge (6:00pm to 8:00am), length of ICU stay and in-hospital death. Diagnosis on ICU admission was categorized as respiratory failure, renal failure, heart failure, disturbance of consciousness, sepsis/infection, post-cardiac arrest, intoxication, severe acute pancreatitis, fulminant hepatic failure, trauma, burn, cardiovascular surgery, other elective surgery, or miscellaneous.

We recorded physiological and laboratory data at ICU discharge as follows: SOFA score, systemic inflammatory response syndrome (SIRS) score [15], white blood cell (WBC) count, serum CRP level, PCT level, blood interleukin-6 (IL-6) level, blood lactate level, serum albumin level, and hemoglobin level. Serum CRP, PCT, and blood IL-6 were ordered after the decision to discharge the ICU patient was made if it had not already been done for clinical reasons. WBC (measurable range, 0.0–10×10^6^/µL; normal range, 4.0–9.0×10^3^/µL) and hemoglobin (measurable range, 0.0–30 g/dL; normal range, 14.0–17.0 [male], 12.0–16.0 [female] g/dL) was measured with an automated hematology system (Sysmex XE-2100; Sysmex Corporation, Kobe, Japan). Serum CRP (measurable range, 0.1–20 mg/dL; normal range <0.2 mg/dL) and albumin (measurable range, 0.1–10 g/dL; normal range, 3.9–5.1 g/dL) were measured with a BioMajesty JCA-BM 8040 automated analyzer (JEOL, Tokyo, Japan) using a CRP-Latex (Ii)X2 assay kit (Denka Seiken Co., Ltd., Tokyo, Japan) and a BCG method (ALB S, Sysmex Corporation, Kobe, Japan), respectively. Serum PCT (measurable range, 0.02–100 ng/mL; normal range, <0.25 ng/dL) and IL-6 (measurable range, 1.5–5000 pg/mL; normal range, <4 pg/mL) were measured with a chemistry analyzer (Cobas e411, Roche Diagnostics GmbH, Mannheim, Germany). Lactate (measurable range, 0.1–20 mmol/L; normal range, <1.3 mmol/L) was measured with a blood gas analyzer (GEM Premier 4000, IL Japan, Tokyo, Japan). When the measurements were out of range, diluted samples were used for analysis.

The primary end point was 90-day mortality after ICU discharge; this was assessed using medical records or through written questionnaires for family members, as was the length of survival and antibiotic use at the time of discharge. Enrolled patients were divided into two groups according to death by 90 day after ICU discharge; survivor group and non-survivor group.

### Statistical Analysis

Data are presented as median (25th–75th percentile), absolute numbers, and percentages. We tested for between-group differences using Fisher's exact test for categorical data and Mann-Whitney's *U* test for continuous data.

To extract factors affecting 90-day mortality, we performed binary logistic regression analysis (stepwise method) using death by 90 day after ICU discharge as a dependent variable. We selected followings as independent variables; age, sex, physiological or laboratory data at ICU discharge that have significant differences between the two groups in Mann-Whitney's U test. Goodness of fit of the model was assessed by the Hosmer-Lemeshow test and calibration plot. Receiver operating characteristic (ROC) analysis was performed among independent variables associated with 90-day mortality after ICU discharge in binary logistic regression analysis., setting the cutoff point to the highest Youden index (sensitivity [%] + specificity [%] − 100) [16]. We also performed a log-rank test of the Kaplan-Meier survival curve derived from the cutoff point of PCT level and SOFA score at ICU discharge and Cox regression analysis (stepwise method) for independent variables associated with 90-day mortality after ICU discharge in binary logistic regression analysis, using survival days after ICU discharge as a survival variable. IL-6 level and PCT level were analyzed by logarithmic transformation. The statistical analysis was performed using SPSS version 22 (IBM Corporation, Chicago, IL) software. Differences were considered significant at *P*<0.05 (two-tailed).

## Results

There were 195 (89.4%) patients in the survivor group, and 23 (10.6%) in the non-survivor group. No significant differences were found for age, and the proportion of male sex was higher in the non-survivor group (**Table 1**). Non-survivors had significantly higher APACHE II and SOFA scores on ICU admission compared to survivors (*P*<0.001). There was no difference in antibiotic treatment between survivors and non-survivors (**Table 1**).

**Table 1:** Baseline characteristics and ICU treatment.

	Survivors (*n* = 195)	Non-survivors (*n* = 23)	*P* value
Age, years	68 (54–75)	70 (63–80)	0.12
Male sex	128 (65.6)	20 (87.0)	0.056
APACHE^a^ II score on ICU^b^ Admission	22.0 (16.0–29.0)	33.0 (25.5–38.0)	<0.001
**Predicted hospital mortality**	**33.3 (18.0**–**67.2)**	**74.0 (51.4**–**88.3)**	**<0.001**
SOFA^c^ score on ICU admission	7.0 (4.0–10.0)	12 (10–15.5)	<0.001
ICU readmission cases	19 (9.7)	4 (17.3)	0.28
Surgical cases	84 (43.1)	4 (17.4)	0.023
Admission diagnosis			
Sepsis/infection	41 (21.0)	8 (34.8)	
Cardiovascular surgery	53 (27.2)	1 (4.3)	
Post-cardiac arrest	11 (5.6)	2 (8.7)	
Intoxication	1 (0.5)	1 (4.3)	
SAP^d^/FHF^e^	6 (3.1)	0 (0)	
Trauma/burn	22 (11.3)	2 (8.7)	
Respiratory failure	18 (9.2)	3 (13.0)	
Renal failure	4 (2.1)	2 (8.7)	
Heart failure	5 (2.6)	0 (0)	
Disturbance of consciousness	6 (3.1)	1 (4.3)	
Miscellaneous	28 (14.4)	3 (13.0)	
Mechanical ventilation	139 (71.3	20 (87.0)	0.14
Renal replacement therapy	49 (25.1)	12 (52.2)	0.012
Vasopressor	85 (43.6)	13 (56.5	0.27
Antibiotics treatment			
During ICU stay	169 (86.7)	19 (78.2)	0.34
At ICU discharge	136 (69.7)	14 (60.9)	0.48
Infection at death	-	14 (60.9)	
Nocturnal ICU discharge	6 (3.1)	1 (4.3)	0.55
ICU length of stay, days	6.0 (4.0–11)	7.0 (4.5–14)	0.15
Inhospital death by 90 day after ICU discharge		17 (73.9)	

Data are medians (25th–75th percentile), n (%), and absolute numbers. *P* values were calculated by Mann-Whitney *U* test or Fisher exact test. Predicted hospital mortality were calculated from APACHE II score.

a APACHE, Acute Physiology and Chronic Health Evaluation score;

b ICU, intensive care unit;

c SOFA, Sequential Organ Failure Assessment score;

d SAP, severe acute pancreatitis;

e FHF, fulminant hepatic failure.

Physiological status and laboratory data at ICU discharge were compared between the survivor and non-survivor groups (**Table 2**). Non-survivors had a higher SOFA score (*P*<0.001) and serum PCT and IL-6 levels (*P*<0.001) and decreased serum albumin (*P* = 0.003) and hemoglobin levels (*P* = 0.004) at ICU discharge compared to survivors.

**Table 2:** Comparison of physiological and laboratory data at ICU discharge between survivors and non-survivors.

Finding at ICU^a^ discharge	Survivors (*n* = 195)	Non-survivors (*n* = 23)	*P* value
SOFA^b^ score	4.0 (3.0–6.0)	8.0 (7.0–10)	<0.001
(a) Respiratory system	1.0 (0–2.0)	2.0 (0–2.0)	0.53
(b) Coagulation	0 (0–2.0)	1 (0–3.0)	0.14
(c) Liver	0 (0–1.0)	2.0 (0–3)	<0.001
(d) Cardiovascular	0 (0–0)	0 (0–0)	0.022
(e) Central nervous system	1.0 (0–2.0)	2.0 (1.0–4.0)	0.001
(f) Renal	0 (0–1.0)	0 (0–3.0)	0.046
SIRS^c^ score	2 (1–3)	2 (2–3)	0.070
(a) BT^d^ (<36°C,>38°C)	38 (19.5)	5 (21.7)	0.78
(b) HR^e^ (>90/min)	107 (54.9)	16 (69.6)	0.19
(c) RR^f^ (>20/min)	168 (86.2)	21 (91.3)	0.75
(d) WBC^g^ (<4,000,>12,000/µL)	78 (40.0)	13 (56.5)	0.179
Laboratory data			
WBC, 10^3^/µL	10.3 (7.5–13.5)	9.2 (5.1–12.6)	0.34
CRP^h^, mg/dL	4.9 (2.2–10.9)	6.8 (3.0–12.1)	0.30
PCT^i^, ng/mL	0.25 (0.12–0.79)	1.23 (0.67–3.21)	<0.001
IL-6^j^, pg/mL	27 (15–59)	70 (33–247)	<0.001
Lactate, mmol/L	0.9 (0.7–1.3)	1.1 (0.9–1.4)	0.065
Albumin, g/dL	2.8 (2.5–3.2)	2.4 (2.1–2.8)	0.003
Hemoglobin, g/dL	9.9 (8.8–11.1)	8.6 (7.9–9.8)	0.004

Data are median (25th-75th percentile), n (%). *P* values were calculated by Mann-Whitney *U* test or Fisher exact test.

a ICU, intensive care unit;

b SOFA, Sequential Organ Failure Assessment;

c SIRS, systemic inflammatory response syndrome;

d BT, body temperature;

e HR, heart rate;

f RR, respiratory rate.

g WBC, white blood cell count;

h CRP, C-reactive protein;

i PCT, procalcitonin;

j IL-6, interleukin-6.

To determine factors associated with 90-day mortality after ICU discharge, binary logistic regression analysis was carried out using age, sex, PCT level, IL-6 level, albumin level, hemoglobin level, and SOFA score as independent variables. Serum PCT level, albumin level, and SOFA score at ICU discharge were significantly associated with altered 90-day mortality after ICU discharge (**Table 3**). Hosmer-Lemeshow test showed chi-square value of 6.96, and P value of 0.54. Calibration plot showed the observed versus the predicted mortality after ICU discharge (**Figure S1**).

**Table 3:** Variables for multiple logistic regression analysis to predict 90-day mortality after ICU discharge.

	OR^a^	95% CI^b^	*P* value
PCT^c^	6.47	2.22–18.9	0.001
Albumin	0.23	0.073–0.725	0.012
SOFA^d^ score	1.60	1.29–1.98	<0.001

Odds ratio associated 1 unit change in each parameter. Hosmer-Lemeshow test showed chi-square value of 6.96, and P value of 0.54.

a OR, odds ratio;

b CI, confidence interval;

c PCT, procalcitonin;

d SOFA, Sequential Organ Failure Assessment.

**Figure 1:**
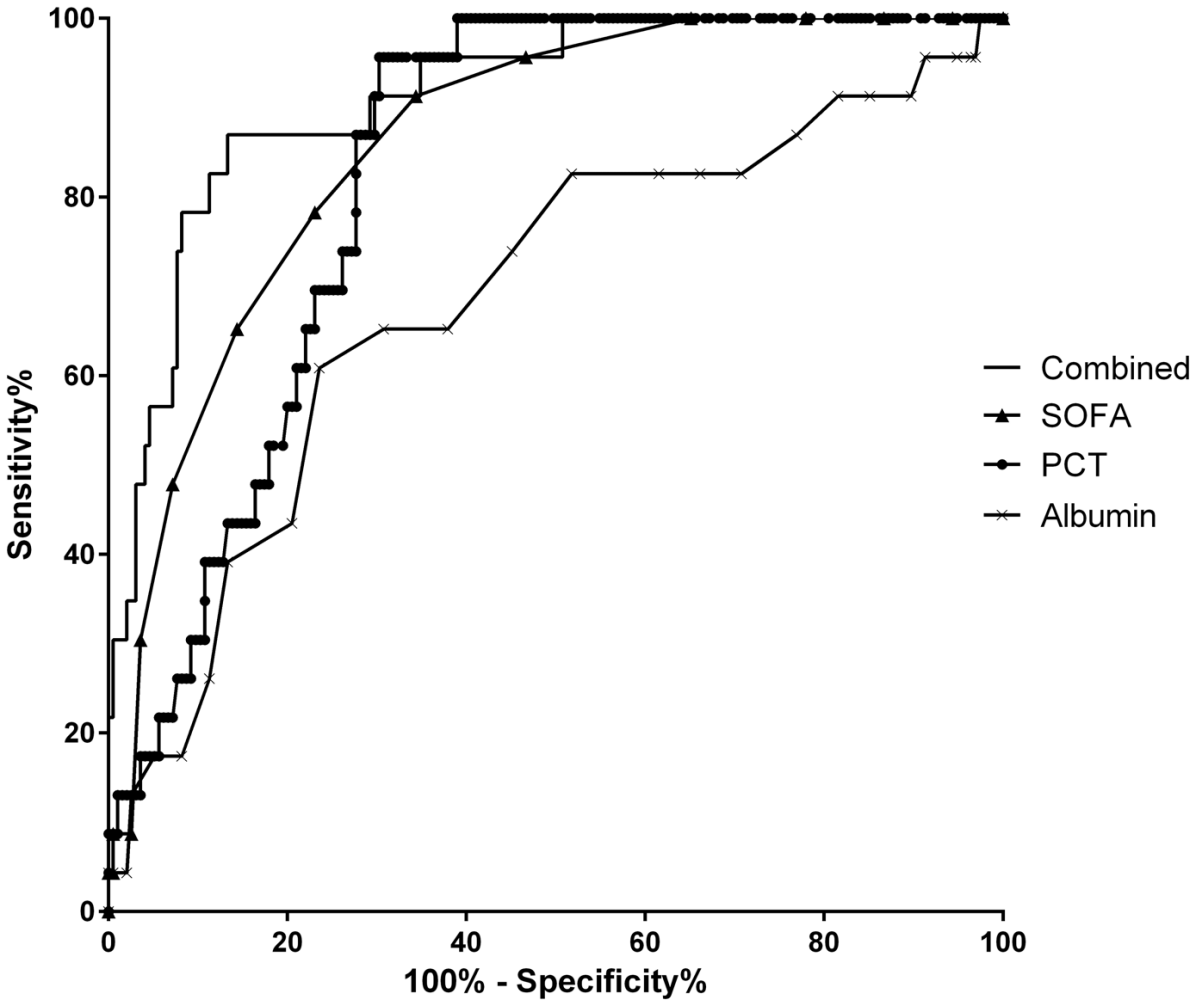
Receiver operating characteristic analysis of SOFA score, serum PCT, and albumin level at ICU discharge and 90-day mortality. The area under the curve (95% confidence interval) was 0.830 (0.771–0.890) for PCT, 0.688 (0.566–0.810) for albumin (inverse), and 0.861 (0.796–0.927) for SOFA score. The AUC for these three indicators combined was elevated to 0.913 (0.858–0.969). ICU, intensive care unit; IL-6, interleukin-6; PCT, procalcitonin; SOFA, Sequential Organ Failure Assessment.

**Figure 2:**
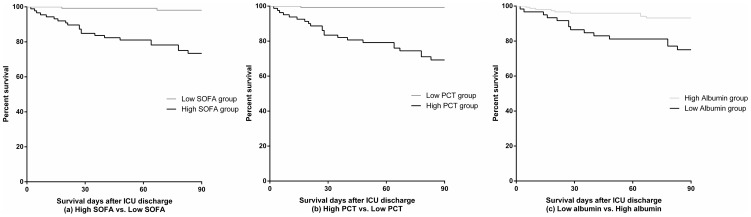
Kaplan-Meier survival curve for 90 days after ICU discharge in high and low groups divided by SOFA, PCT, and albumin. (a) High SOFA vs. Low SOFA group. (b) High PCT vs. Low PCT group. (c) Low albumin vs. High albumin group. The graph shows curves for the two groups (high SOFA vs. low SOFA, high PCT vs. low PCT, and low albumin vs. high albumin) derived from the cutoff value for SOFA score at 6 (*P*<0.001), PCT level at 0.57 ng/mL (*P*<0.001), and albumin level at 2.5 g/dL (*P* = 0.001) at ICU discharge (log-rank test). ICU, intensive care unit; PCT, procalcitonin; SOFA, Sequential Organ Failure Assessment.

We performed a ROC analysis to determine the predictive level of serum PCT, albumin level, and SOFA score at ICU discharge for 90-day mortality after ICU discharge. The cutoff value to achieve the highest Youden index was 0.57 ng/mL for PCT (sensitivity, 95.7%; specificity, 69.7%), 2.5 g/dL for albumin level (sensitivity, 60.9%; specificity, 76.4%), and 6 for SOFA score (sensitivity, 91.3%; specificity, 65.6%). The area under the curve (AUC) for each factor was 0.830 (95% confidence interval [CI], 0.771–0.890) for PCT, 0.688 (0.566–0.810) for albumin, and 0.861 (0.796–0.927) for SOFA score, respectively (**Figure 1**). The AUC for the combination of PCT level, SOFA score, and albumin level increased to 0.913 (0.858–0.969). Groups with high SOFA, high PCT, or low albumin had a significantly higher 90-day mortality rate after ICU discharge in Kaplan-Meier survival analysis (log-rank test) (**Figure 2**). We also performed Cox regression analysis using serum PCT, albumin level, and SOFA score at ICU discharge as independent variables, survival days after ICU discharge as survival variable. Serum PCT and SOFA score were significantly associated with survival days after ICU discharge (**Table 4**).

**Table 4:** Variables for Cox regression analysis associated with survival days after ICU discharge.

	HR^a^	95% CI^b^	*P* value
PCT^c^	3.87	2.20–8.81	<0.001
SOFA^d^ score	1.22	1.11–1.24	<0.001

Hazard ratio associated 1 unit change in each parameter.

a HR, hazard ratio;

b CI, confidence interval;

c PCT, procalcitonin;

d SOFA, Sequential Organ Failure Assessment.

## Discussion

This study was conducted to identify prognostic factors associated with 90-day mortality after ICU discharge. The results indicate that non-survivors present a higher SOFA score, higher serum PCT and IL-6 levels, and lower serum albumin and hemoglobin levels at ICU discharge compared to survivors. Subsequent logistic regression analysis showed that serum PCT level, albumin level, and SOFA score at ICU discharge were associated with 90-day mortality after ICU discharge. While individual AUC for SOFA score, PCT level, and albumin level presented high diagnostic accuracy, the accuracy increased when these factors were combined; this could be useful for decisions regarding ICU discharge.

Serum CRP level at ICU discharge [9], [10], a reduction of CRP level>25% in the last 24 hours before ICU discharge [17], CRP/albumin ratio [11], and heart rate at ICU discharge [12] have been reported as useful predictive markers or indicators of long-term outcome. On the other hand, reports have indicated that CRP levels at ICU discharge were not a predictor of hospital mortality [18], [19]. In the present study, CRP level; any SIRS criterion, including heart rate; or the total SIRS score were not predictive of mortality after ICU discharge (**Table 2**). This might be because CRP and SIRS subscores are markers that may be elevated in inflammation without infection.

Blood PCT level shows a significant rise in cases of bacterial infections but is suppressed by interferon-γ in cases of viral infections. This characteristic elevates the sensitivity for bacterial infections [20]–[22]. PCT has also been known to resist suppression by administration of steroids [23] compared with other biomarkers. Owing to these characteristics, elevated PCT level at ICU admission has been reported as a good diagnostic marker for patients with suspected sepsis [24], [25]. Thus, PCT has better diagnostic accuracy for sepsis than CRP or IL-6 [24],[26]. Although its diagnostic value for acute infection is not as high as IL-6, PCT is expected to be a highly specific marker for chronic and persistent infection occurring in late phase sepsis [27]. There was a report on the predictive value of a PCT decrease in first 72 hours in the ICU for hospital mortality [28], but the present study is the first to report that PCT at discharge predicts post-ICU mortality. This study analyzed physiological status and laboratory data at ICU discharge, which is simpler than maximum value or sequential measurement of a certain biomarker during the ICU stay, and is more applicable to the clinical setting. Patients without sepsis or infection on ICU admission may also be treated with antibiotics because of complicated or nosocomial infection. Therefore, no significant differences were observed in the proportion of antibiotics treatment during ICU stay between survivors and non-survivors. However, incidence of infection at death in non-survivors was 60.9%, which is the same as the proportion of antibiotics treatment at ICU discharge. It can be speculated that PCT level reflected persistent infection, even when no significant differences were seen in the proportion of antibiotics treatment at ICU discharge between survivors and non-survivors.

The SOFA score indicates organ dysfunction and is widely used as a severity measure for critically ill patients. Persistent organ dysfunction might affect post-ICU mortality. Previous studies showed that SOFA score on ICU admission or maximum SOFA score during the ICU stay was predictive of ICU or hospital mortality [29], [30], but no study has reported an association of SOFA score at discharge and post-ICU mortality. This is the original perspective of the present study.

Serum albumin levels are widely used to assess the general nutritional status of patients [31]. Low serum albumin levels have been reported to be associated with post-ICU mortality [18], [32]. The present results showed that low serum albumin is an independent risk factor for 90-day mortality after ICU discharge. Persistent inflammation may have led to malnutrition of the study patients [33].

In the present study, serum PCT level (AUC, 0.830) and SOFA score (AUC, 0.861) accurately predicted 90-day mortality after ICU discharge. Each parameter itself had accurate diagnostic ability. Furthermore, combining PCT and albumin levels with SOFA score elevated the AUC to as high as 0.913 (95% CI, 0.858–0.945). However, when patients fall into unresolvable coma or renal failure, the SOFA score is persistently elevated, making it inappropriate as an indicator for deciding discharge from the ICU. Moreover, SOFA score does not include parameters of infection or malnutrition, diagnostic accuracy for post-ICU mortality was improved when combining SOFA score with serum PCT level (an indicator of infection) and serum albumin level (an indicator of malnutrition). These parameters can be appropriate criteria for ICU discharge.

Some patients discharge from the ICU even though they do not fulfill the criteria above. We could not evaluate the efficacy of "step-down” unit because our hospital does not have it. However, it will certainly play important roles in such patients.

This study has several limitations. First, it was a single-center observational study, so ICU physician and ward physician were not blinded to the study blood test. Treatment of the patients may have been changed by the results of the laboratory examination. Especially, albumin and hemoglobin levels can be elevated by replacement or transfusion therapy. This may have resulted in treatment bias. Second, because mortality after ICU discharge was very low, the results may not be applicable to centers with higher mortality rates. Third, some non-survivors died other hospital and we did not collected post-mortem data. We could not assess the post ICU death were preventable or not.

## Conclusions

Non-survivors presented a higher SOFA score and serum PCT and IL-6 levels, as well as lower serum albumin and hemoglobin levels at ICU discharge. Serum PCT level, albumin level, and SOFA score at ICU discharge were independently associated with 90-day mortality after ICU discharge. High serum PCT level (≧0.57 ng/mL) and SOFA score (≧6) predict post-ICU mortality and survival days after ICU discharge, and the combination of these two parameters with albumin level might enable accurate prediction of post-ICU mortality.

## Supporting Information

Figure S1 **Calibration plot showing the observed probability versus the predicted mortality.** Goodness of fit of the model was assessed by the Hosmer-Lemeshow test; chi-square value of 6.96, P value of 0.54. The diagonal line indicates perfect calibration (predicted mortality equal observed mortality).
(PDF)
