# Supplementary figures and images for: Serum Procalcitonin Level and SOFA Score at Discharge from the Intensive Care Unit Predict Post-Intensive Care Unit Mortality: A Prospective Study
Source: PLoS One. 2014 Dec 2;9(12):e114007. doi: 10.1371/journal.pone.0114007 (PMC4252062; doi:10.1371/journal.pone.0114007)

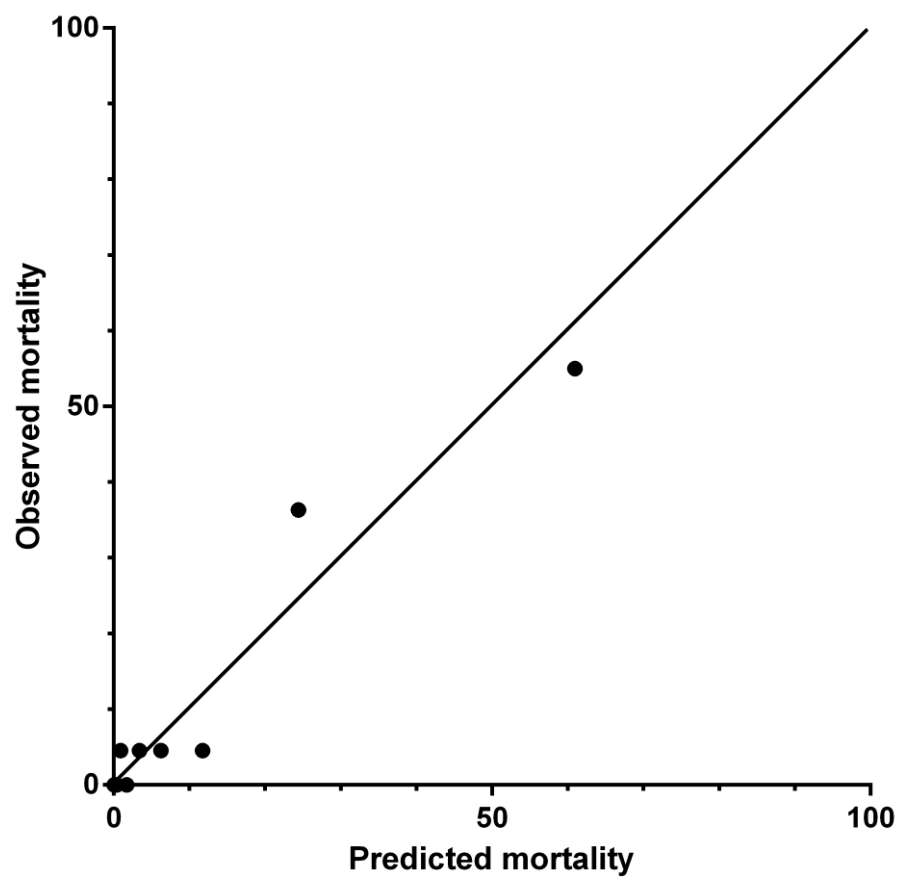

Supplement: Figure S1 — Calibration plot showing the observed probability versus the predicted mortality. Goodness of fit of the model was assessed by the Hosmer-Lemeshow test; chi-square value of 6.96, P value of 0.54. The diagonal line indicates perfect calibration (predicted mortality equal observed mortality). (PDF) [file pone.0114007.s001.pdf]
